# External quality assessment of orthohantavirus and lymphocytic choriomeningitis virus molecular detection and serology in Europe, 2021

**DOI:** 10.2807/1560-7917.ES.2023.28.40.2300054

**Published:** 2023-10-05

**Authors:** Mert Erdin, Kamelia R Stanoeva, Ramona Mögling, Miša Korva, Nataša Knap, Katarina Resman Rus, Cristina Domingo, Johan HJ Reimerink, Ankje de Vries, Hussein Alburkat, Mira Utriainen, Céline M Gossner, Tarja Sironen, Tatjana Avšič-Županc, Chantal BEM Reusken, Olli Vapalahti

**Affiliations:** 1 Department of Virology, Medicum, Faculty of Medicine, University of Helsinki, Helsinki, Finland; 2 Centre for Infectious Disease Control, National Institute for Public Health and the Environment (RIVM), Bilthoven, the Netherlands; 3 Institute of Microbiology and Immunology, Faculty of Medicine, University of Ljubljana, Ljubljana, Slovenia; 4 Centre for Biological Threats and Special Pathogens, Robert Koch Institute (RKI), Berlin, Germany. Current affiliation: Centre for International Health Protection, RKI, Berlin, Germany; 5 Diseases Programme Unit, European Centre for Disease Prevention and Control (ECDC), Solna, Sweden; 6 Department of Veterinary Biosciences, Faculty of Veterinary Medicine, University of Helsinki, Helsinki, Finland; 7 Helsinki University Hospital Diagnostic Center, HUSLAB, Helsinki, Finland; * These authors contributed equally to the work and share the first authorship.; ** These authors contributed equally to the work and share the last authorship.

**Keywords:** Hantaviruses, Lymphocytic choriomeningitis virus, External Quality Assessment, molecular diagnostics, serology, Europe

## Abstract

**Background:**

Rodent-borne viruses such as orthohantaviruses and arenaviruses cause considerable disease burden with regional and temporal differences in incidence and clinical awareness. Therefore, it is important to regularly evaluate laboratory diagnostic capabilities, e.g. by external quality assessments (EQA).

**Aim:**

We wished to evaluate the performance and diagnostic capability of European expert laboratories to detect orthohantaviruses and lymphocytic choriomeningitis virus (LCMV) and human antibody response towards orthohantaviruses.

**Methods:**

We conducted an EQA in 2021; molecular panels consisted of 12 samples, including different orthohantaviruses (Seoul, Dobrava-Belgrade (DOBV), Puumala (PUUV) and Hantaan orthohantavirus), LCMV and negative controls. Serological panels consisted of six human serum samples reactive to PUUV, DOBV or negative to orthohantaviruses. The EQA was sent to 25 laboratories in 20 countries.

**Results:**

The accuracy of molecular detection of orthohantaviruses varied (50‒67%, average 62%) among 16 participating laboratories, while LCMV samples were successfully detected in all 11 participating laboratories (91-100%, average 96%). The accuracy of serological diagnosis of acute and past orthohantavirus infections was on average 95% among 20 participating laboratories and 82% in 19 laboratories, respectively. A variety of methods was used, with predominance of in-house assays for molecular tests, and commercial assays for serological ones.

**Conclusion:**

Serology, the most common tool to diagnose acute orthohantavirus infections, had a high accuracy in this EQA. The molecular detection of orthohantaviruses needs improvement while LCMV detection (performed in fewer laboratories) had 95% accuracy. Further EQAs are recommended to be performed periodically to monitor improvements and challenges in the diagnostics of rodent–borne diseases.

**Key public health message**

**What did you want to address in this study?**

Emerging viruses newly appear or increase in frequency and may cause outbreaks or even pandemics. Orthohantaviruses and lymphocytic choriomeningitis virus, carried by rodents, are of public health concern as emerging viruses. We (Emerging Viral Disease Expert Laboratory Network) aimed to evaluate how well the European expert laboratories can detect these viruses.

**What have we learnt from this study?**

Detection of orthohantavirus genetic material by molecular methods requires improvement. Lymphocytic choriomeningitis virus molecular detection is relatively good. Detecting orthohantavirus infections by measuring human antibodies raised against them is at satisfactory level, especially for diagnosing acute cases.

**What are the implications of your findings for public health?**

Orthohantaviruses and lymphocytic choriomeningitis virus are important causes for possibly preventable human infections. Being able to accurately diagnose them may help general surveillance, early detection of outbreaks, and localisation of risk areas, where the infections occur. That in turn can lead to better and timely public health measures and prevention of these diseases.

## Introduction

Rodent-borne viruses, such as orthohantaviruses and arenaviruses, cause considerable disease burden with regional and temporal differences in incidence and clinical awareness and present potential for outbreaks. Spillover events between various rodent species or from host rodents to humans are frequent [1], and preparedness is essential. One of the main aspects of preparedness for sporadic infections or outbreaks is the ability to diagnose them efficiently and accurately, and hence diagnostic methods need to be evaluated regularly. While two external quality assessments (EQAs) have been performed on orthohantavirus serology in Europe in the past 20 years [2,3], no EQA on molecular diagnostics has been performed for neither orthohantaviruses nor for lymphocytic choriomeningitis virus (LCMV).

Orthohantaviruses (genus Orthohantavirus, family Hantaviridae) are widely distributed in Eurasia. Dobrava-Belgrade orthohantavirus (DOBV), Seoul orthohantavirus (SEOV) and Hantaan orthohantavirus (HTNV) cause haemorrhagic fever with renal syndrome, while Puumala orthohantavirus (PUUV) causes a milder form called nephropathia epidemica [4]. Distribution of these clinically important orthohantaviruses varies partly depending on the reservoir host distribution. The main reservoir hosts of the orthohantaviruses in Europe are yellow-necked mice (*Apodemus flavicollis,* distributed throughout most of Europe) and striped field mice (*Apodemus agrarius*, Central and Eastern Europe) for different lineages of DOBV, black rats and brown rats (*Rattus rattus/norvegicus*, throughout the globe) for SEOV and bank voles (*Myodes glareolus*, throughout Europe) for PUUV [4-6]. Transmission of orthohantaviruses to humans may occur through inhalation of virus-containing aerosols of rodent excreta or direct contact with the reservoir hosts [4]. In 2020, 1,647 cases [7] of orthohantavirus infections were notified in the European Union/European Economic Area (EU/EEU) countries, most of them in Finland (1164 cases), Germany (229 cases) and Sweden (61 cases). In the light of overlapping distribution of host reservoirs, differences in clinical severity, heterologous vs homologous antibody responses and epidemiological patterns, it is valuable to identify the species of the causative orthohantavirus.

Lymphocytic choriomeningitis virus (genus Mammarenavirus, family Arenaviridae) is an arenavirus with emerging potential due to the worldwide distribution of its host, the house mouse (*Mus musculus*) [8]. Transmission to humans occurs via inhalation of house mouse secreta (mostly nasal secretions) or direct contact with the infected host animal [8]. The clinical presentation varies from an asymptomatic infection to severe neurological and congenital disease [9-11]. Because of the variety of symptoms and possibly high proportion of mild or asymptomatic infections, detection of LCMV and diagnosis of the infection in humans are seldom considered and therefore underdiagnosed [8]. Thus, it is of importance to demonstrate the use of and assess the diagnostic methods.

The aim of this study was to evaluate the performance and diagnostic capability of European expert laboratories (in the EU/EEA countries and the EU pre-accession countries) to detect orthohantaviruses and LCMV with molecular approaches and human antibody response towards orthohantaviruses by performing an EQA.

## Methods

### Participants

In August 2021, member laboratories of the European Emerging Viral Diseases Expert Laboratory Network (EVD-LabNet) were invited to register for this EQA or to forward the invitation to competent laboratories in their countries. A choice between receiving a molecular and serological panel, or both, was offered upon EQA registration.

### Preparation of the molecular panel

The molecular panel consisted of 12 samples: seven samples with four different orthohantaviruses (one sample with HTNV, two samples with SEOV, two samples with DOBV and two samples with PUUV), two samples with LCMV and three samples were negative. The panel composition is presented in Table 1. Virus stock solutions were prepared by growing LCMV Armstrong (007v-EVA02708), HTNV 76-118 (007v-EVA02761), SEOV R22 (007v-EVA00922), DOBV 907/5 (007v-EVA00807) and PUUV Cg18-20 (007v-EVA00809) in Vero E6 cells (African green monkey epithelial cells, clone E6) in Dulbecco’s modified Eagle’s medium with high glucose and GlutaMAX supplement (Thermo Fisher Scientific, 61965026), supplemented with 4% FBS (Euroclone, ECS0180L) at 37°C, 5% CO_2_ atmosphere and 95% relative humidity. Viruses were grown for 7 (LCMV), 13 (HTNV), 14 (SEOV and DOBV) and 15 days (PUUV), respectively. All cell culture experiments were performed in Biosafety Level 3 facilities. After incubation, the cell culture supernatants were collected and centrifuged twice at 4°C (10 min at 3200 × g and 5 min at 20,800 × g) in an Eppendorf 5804R centrifuge (Eppendorf, Hamburg, Germany). Supernatants containing viruses were heat and gamma irradiation inactivated (60°C, 1 h). After inactivation, final viral stock quantification was performed as described below. Undiluted viral concentrations were: HTNV 7.10 × 10^2^ copies/mL, SEOV 1.90 × 10^3^ copies/mL, PUUV 4.80 × 10^3^ copies/mL, DOBV 1.70 × 10^4^ copies/mL and LCMV 2.40 × 10^5^ copies/mL. Subsets of the inactivated supernatant were diluted as indicated in Table 1.

**Table 1:** Orthohantavirus and lymphocytic choriomeningitis virus molecular panel composition and performance of participating laboratories (n = 16) of external quality assessment, Europe, 2021

Sample ID	Virus	Strain	GenBank	Dilution	Submissions	Laboratories	Correct results			False negative	Inconclusive
							Submissions		Laboratories		
							n	%			
1	HTNV	76-118	NC005222	Undiluted	17	13	11	65	9	5	1
4	SEOV	R22	AF488707	Undiluted	15	14	10	67	8	4	1
9	SEOV	R22	AF488707	Undiluted	15	14	11	73	9	3	1
6	DOBV	907/5	L41916^a^	Undiluted	18	14	8	44	8	9	1
7	DOBV	907/5	L41916^a^	1:10	18	14	7	39	7	9	2
10	PUUV	CG1820	M63194	Undiluted	20	15	11	55	10	9	0
12	PUUV	CG1820	M63194M	1:10	20	15	10	50	10	80	2
3	LCMV	ARM 53b	M20869	1:10	11	11	11	100	11	0	0
8	LCMV	ARM 53b	M20869	1:1000	11	11	10	91	11	1	0
Controls (n = 3)	NEG	Not applicable			153	16	135	88	16	2^b^	16

DOBV: Dobrava-Belgrade orthohantavirus; HTNV: Hantaan orthohantavirus; ID: identification; LCMV: lymphocytic choriomeningitis virus; NEG: negative; PUUV: Puumala orthohantavirus; SEOV: Seoul orthohantavirus.

^a^The DOBV 907/5 isolate, used in this EQA gives 99-100% nucleotide similarity with the DOBV 3970/87 isolate, submitted to GenBank.

^b^False positive reported for negative control samples.

The molecular EQA panel was tested by two independent laboratories before distribution to the participants. For detection of the orthohantaviruses, one expert laboratory used multiple S-segment targeting real-time reverse transcription (RT)-PCR assays [12,13] while the other expert laboratory used the RT-PCR methods as described by Kramski et al. [13]. The two expert laboratories tested detection of LCMV by an in-house S-segment targeting real-time PCR, developed on the basis of alignment of different LCMV S-segment sequences. Briefly, the reaction mix consisted of TaqManFast Virus 1-Step Master Mix (Applied Biosystems), 0.2 µM primers (LCMV S: 5'-gggATCCTAggCTTTTTggAT-3' and LCMV As: 5'-gCCAATAATgACAATZgTTgAT-3') and 0.3 µM probe (LCMV P 5'-FAM-CCTCAAACATTgTCACAATCTgACCCAT-TAMRA-3') used with the following temperature protocol: 50°C for 5 min, 95°C for 20 s, 40 cycles at 95°C for 3 s and 60°C for 30 s. Testing by the expert laboratories yielded consistent results.

### Preparation of the serology panel

The serology panel consisted of six human serum samples. Two samples were reactive to PUUV, two to DOBV with commercial enzyme immunoassay (Reagena, Toivala, Finland) and two negative control samples did not contain reactive antibodies against orthohantaviruses. All samples were heat inactivated (56°C, 1 h) before aliquoting. The panel composition is presented in Table 2. For each virus, a sample with both IgM and IgG antibodies, thus representing an acute infection and a sample with IgG but no IgM antibodies, representing a past infection, were included. Prior to shipping to participants, two expert laboratories tested the serology panel using both commercial enzyme immunoassays and in-house immunofluorescence assays. One expert laboratory used both a commercial enzyme immunoassay for DOBV and PUUV (Reagena, Toivala, Finland) and an in-house immunofluorescence assay [14]. The other expert laboratory used a commercial indirect immunofluorescence assay (Hantavirus Mosaic 1 biochips, Euroimmun, Lübeck, Germany).

**Table 2:** Orthohantavirus serology panel composition and performance of participating laboratories (n = 20) of external quality assessment, Europe, 2021

Sample ID	Virus	Infection phase	Antibody type	Submissions	Correct results	False results	Inconclusive results	Cross-reactions^a^	Sensitivity (%)^b^
1	PUUV	Past infection	IgM-/IgG+	20	12	2	3	3	75
3	DOBV	Acute infection	IgM+/IgG+	19	14	0	1	4	95
4	DOBV	Past infection	IgM-/IgG+	19	12	0	2	5	90
6	PUUV	Acute infection	IgM+/IgG+	20	17	1	0	2	95
2	NEG	Not applicable		55	55	0	0	0	100
5	NEG	Not applicable		55	55	0	0	0	100

DOBV: Dobrava-Belgrade orthohantavirus; ID: identification; NEG: negative; PUUV: Puumala orthohantavirus.

^a^These cross-reactive results were one of the following: heterologous detections of different sero-group orthohantaviruses, homologous detection of same sero-group but different orthohantavirus species, between acute infection (IgM + /IgG + ) and past infection (IgM−/IgG + ) samples.

^b^Sensitivity (%) calculated by using both total number of correct results and total number of cross-reaction results.

Unfortunately, we could not produce serology panel samples for LCMV due to shortage of available sample material necessary for testing of the panel and supply for all participating laboratories.

### Distribution of the panels

All samples of the molecular panel were freeze-dried, and serum samples of the serology panel were lyophilised. The EQA was dispatched at ambient temperature in November 2021 to the EQA participants, including accompanying instructions and if requested, customs documentation.

### Result submission and evaluation

We collected EQA results via online submission forms (separate molecular and serology forms), hosted on the EU-survey platform. Participants received instructions and links to access the forms via email. Brief instructions were also provided within the results submission forms. We asked participants to submit methodological information, outcomes of each assay used for the diagnosis and the final (diagnostic) result per panel sample. The results for the molecular and serology panel were analysed separately. We used the number of correct results in the total number of samples in each panel to calculate the accuracy.

Microsoft Excel Office 365 and R versions 4.1.3 to 4.3.0/R studio were used for data processing and analysis. Maps were created using the ECDC Map Maker tool (EMMa).

## Results

### Participation in the external quality assessment

**Figure 1:**
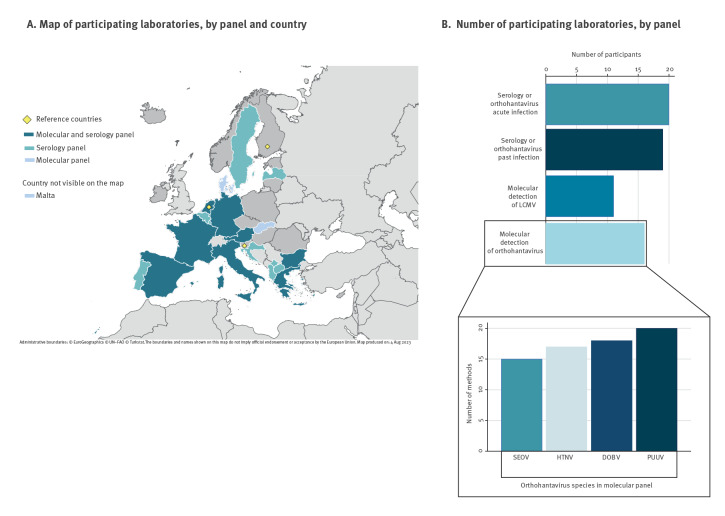
Participating laboratories of the external quality assessment of orthohantaviruses and lymphocytic choriomeningitis virus, Europe, 2021 (n = 25)

DOBV: Dobrava-Belgrade orthohantavirus; HTNV: Hantaan orthohantavirus; LCMV: lymphocytic choriomeningitis virus; PUUV: Puumala orthohantavirus; SEOV: Seoul orthohantavirus.

In total, twenty-five laboratories from 20 EU countries or EU pre-accession countries participated in the EQA. Seventeen laboratories opted to receive both panels, while five and three laboratories requested only the molecular or serology panel respectively. In terms of submitted EQA responses, sixteen laboratories (out of 22 received panels) participated in the molecular panel (Figure 1A). All these 16 laboratories used one or more orthohantavirus-specific detection methods, and 11 laboratories used a molecular assay targeting LCMV (Figure 1B). Assays targeting PUUV showed the highest number of different methods, while SEOV had the lowest number.

For molecular detection of LCMV, all participating laboratories used one method. The lower part of the graph represents the number of methods used by participant laboratories for each orthohantavirus species.

Twenty of the 25 laboratories (all sent out serology panels) participated in the serology panel (Figure 1A). All these 20 laboratories used IgM assays for detecting acute infection (IgM and IgG positive) and there were a total of 27 submissions of IgM results, while 19 laboratories tested the panel samples also for previous or recent infection (positive IgG and negative IgM), adding up to a total of 27 submissions of IgG results from these 19 laboratories (Figure 1B).

### Performance of participant laboratories

**Figure 2:**
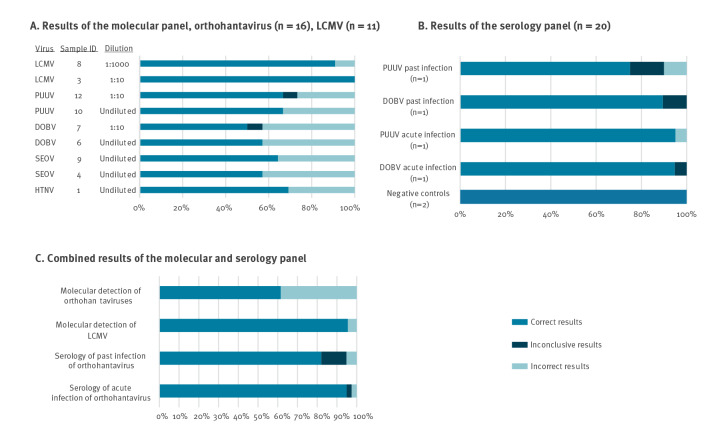
Results of detection of orthohantaviruses and lymphocytic choriomeningitis virus and 2021 EQA in Europe

DOBV: Dobrava-Belgrade orthohantavirus; HTNV: Hantaan orthohantavirus; LCMV: lymphocytic choriomeningitis virus; PUUV: Puumala orthohantavirus; SEOV: Seoul orthohantavirus.

The proportion of correct results for molecular detection of orthohantaviruses varied between 50% and 67% and between 91% and 100% for LCMV (Figure 2A). The accuracy was around 62% for orthohantaviruses and 96% for LCMV (Figure 2C). Accuracy corresponds to the capability of laboratories to give right diagnosis to each sample (Table 1).

Accuracy of orthohantavirus serology for acute infection was 95%, while it was around 82% for past infection (Figure 2C, Table 2). While the accuracy of the serology for acute orthohantavirus infection of DOBV and PUUV was 95%, the accuracy for past infection was 75% for PUUV and 90% for DOBV (Figure 2B). The results for each sample are presented in Table 2.

### Results of the molecular panel

Eight different automated systems were used for nucleic acid extraction by 10 laboratories and three different manual nucleic acid extraction methods by six laboratories. More details on the methods for nucleic acid extractions can be found in Supplementary Table S3.

All participants of the molecular EQA tested the panel samples for at least one orthohantavirus species. There were 23 submissions from 16 laboratories with some laboratories using and reporting results of more than one method (Table 3): 16 laboratories used published in-house assays, three laboratories used commercial assays and seven laboratories used unpublished in-house or own-design assays. There was further variety in which orthohantavirus and which segment (mostly L and S) the submitted methods targeted. The total percentage of correct positive results of all applied methods for detection of orthohantaviruses was 59% (Table 3). The detailed list of orthohantavirus molecular methods used and results reported is available in Supplementary Table S1.

**Table 3:** Results and summary of methods used for detection of orthohantaviruses and lymphocytic choriomeningitis virus in the molecular panel of the external quality assessment, Europe, 2021

Method	Number of laboratories	Target	Correct positive /expected positives^a^	False negative/expected positives^a^	False positive/expected negatives^a^	Inconclusive^a^
Orthohantaviruses (7 samples)						
In-house PCR [12,13,15-20]	5	Pan-Hanta L-segment	9/35	26/35	0/25	1/60
	3	Hantavirus S-segment	14/21	7/21	0/15	2/36
	12	Virus-specific S-segment	28/35	6/35	4/109	3/144
	3	Virus-specific unknown gene target^b^	9/10	0/10	0/26	1/36
Commercial PCR assays	2	HTNV	0/1	1/1	2/11	0/12
		Hantavirus generic unknown gene target	5/7	2/7	0/5	0/12
Sequencing Illumina NextSeq	1	Unbiased HTS approach	3/7	4/7	0/5	0/12
Total (%)			58.62	39.66	3.06	2.24
Lymphocytic choriomeningitis virus (2 samples)						
In-house PCR [21-25]	1	Pan-Arena L-segment	2/2	0/2	0/10	0/12
	6	LCMV S-segment	12/12	0/12	0/60	0/72
	2	LCMV L-segment	3/4	1/4	2/20	0/24
	1	LCMV unknown gene target^b^	2/2	0/2	0/10	0/12
Sequencing	1	LCMV sequencing	2/2	0/2	0/10	0/12
Total (%)			95.45	4.55	1.81	0

HTNV: Hantaan orthohantavirus; HTS: high-throughput sequencing; LCMV: lymphocytic choriomeningitis virus.

^a^Correct positive results and false negative were calculated by taking into account the number of panel samples containing the target virus. In contrast, inconclusive results and false positive were calculated using the total number of samples (n = 12) and expected number of negative samples. The number of expected results varies depending on the used methods.

^b^Laboratories that did not provide any protocol for molecular detection were classified under unknown.

Eleven of 16 laboratories participated in the LCMV molecular panel (Table 3). Among the 11 laboratories testing for LCMV, five used in-house assays previously published, one used sequencing, one an unpublished own-design molecular assay and four did not give information on the method applied. Assays targeting LCMV S-segment were used by five laboratories and L-segment by three. The percentage of correct positive results reported for LCMV was 95% (Table 3). The detailed list of LCMV molecular methods used and results reported is available in Supplementary Table S1.

### Results of the serology panel

Thirteen laboratories used enzyme immunoassays (EIA) for IgM testing, six used immunoblotting (IB) and four used both immunochromatography (IC) and immunofluorescence assays (IFA). While EIAs targeted generic orthohantaviruses or orthohantavirus antigenic pools, IBs, ICs and IFAs targeted specific orthohantavirus species. The percentage of correct results reported for serology of acute orthohantavirus infection was 94% (at assay level). The proportion of cross-reactions, defined as a positive result between orthohantaviruses from different serogroups or an IgM negative/IgG positive sample reported IgM positive, was 15% (Table 2, Table 4). More details can be found in Supplementary Table S2. Testing for IgG was mainly performed by EIA: 13 of the 19 participating laboratories. Eight laboratories used IFA and six used IB. As with IgM assays, IgG assays also targeted orthohantavirus antigen pools in EIAs and specific orthohantavirus species in IFAs and IBs. In total, 91% of the results for serology of past orthohantavirus infection were correct (at assay level). The total proportion of cross-reactions, meaning a positive result caused by orthohantaviruses from a different serogroup (PUUV-like vs. DOBV-like), or a heterologous virus within the group (DOBV vs SEOV) was 11% (Table 4). A detailed list of serology methods used and results reported is given in Supplementary Table S2.

**Table 4:** Results and summary of methods for determination of acute and past infection of orthohantaviruses in the serology panel of the external quality assurance, Europe, 2021

Method	Assays	Number of laboratories	Target	Correct results^a^	False-negative results^a^	Cross-reactive results^a,b^	Inconclusive results^a^
Acute infection (IgM)							
EIA	Commercial	1	Hantavirus sero-group or virus specific	1/1	0/1	0/4	0/4
		11	Hantavirus generic	21/22	1/22	8/44	9/44
	In-house [26]	1	Hantavirus sero-group or virus specific	2/2	0/2	1/4	0/4
ICA	Commercial	4		4/4	0/4	3/16	2/16
IB	Commercial	6		12/12	0/12	1/24	4/24
IFA	Commercial	3		4/6	0/6	3/12	2/12
	In-house	1		2/2	0/2	0/4	0/4
Total (%)				93.87	2.04	14.81	15.74
Past infection (IgG)							
EIA	Commercial	1	Hantavirus sero-group or virus specific	2/2	0/2	0/4	0/4
		11	Hantavirus generic	41/44	1/44	0/44	2/44
	In-house [25]	1	Hantavirus sero-group or virus specific	2/2	0/2	2/4	0/4
IB	Commercial	6		22/24	0/24	2/24	0/24
IFA	Commercial	3		8/12	0/12	2/12	2/12
	In-house	5		9/10	0/10	6/20	1/16
Total (%)				89.36	1.06	11.11	4.80

EIA: enzyme Immunoassay; IB: immunoblot assay; ICA: immunochromatography assay; IFA: immunofluorescence assay.

^a^The number of correct results, inconclusive results and false-negative results were calculated using only the total number of target samples (n = 91). In contrast, cross-reactive results were calculated by including all samples that might potentially give a positive result (n = 108; number of submissions × number of potentially reactive samples for one submission = total number of potentially reactive samples).

^b^These cross-reactive results were either heterologous detections of different sero-group orthohantaviruses or between acute infection (IgM + /IgG + ) and past infection (IgM-/IgG + ) samples.

## Discussion

The results of the serological part of this EQA performed among 20 expert laboratories in Europe, suggest that serological diagnostics for acute orthohantavirus infection was performed with 95% accuracy. Serological methods are the most common means to diagnose an orthohantavirus infection in Europe. However, the molecular part of this EQA showed that sensitivity of the molecular diagnostics for orthohantaviruses should be improved.

In the molecular EQA for orthohantaviruses, correct results were obtained in 62% of positive samples. One reason for this suboptimal performance might be the widespread use of pan-hantavirus targeting primers [15]; the molecular detection sensitivity for orthohantaviruses of these primers was low. Even though this method shows good performance for higher viral loads and is particularly useful for detection of new or previously unknown orthohantaviruses from rodents, it did not provide good results for diagnostic purposes in this EQA. Interestingly, the study also suggests that methods targeting S-segment instead of L-segment of orthohantaviruses performed better.

Even though LCMV cases might be unrecognised or unreported, the LCMV molecular EQA gave 96% correct results. Similar to orthohantaviruses, LCMV S-segment is the one target that participants preferred. One false negative detection for LCMV was from a method targeting the L-segment. However, notably, not all participating laboratories tested for LCMV (11 out of 16) and given the global and pan-European circulation of LCMV, all expert laboratories should be alert about LCMV presence and have the capability to diagnose LCMV infections.

Most of the serological methods for acute infection used were commercial assays, that in general lack the ability to differentiate between responses to orthohantaviruses at the species level. Such differentiation is challenging unless laboratories use species-specific assays or use molecular methods in parallel. However, knowledge of the orthohantavirus species may not be essential for the clinical diagnosis as long as orthohantavirus infection is confirmed and both PUUV and DOBV-related antigens are used to cover the serological spectrum in Europe. The assays were accurate yielding correct results in 94% of positive samples. In a previous EQA from 2012, the percentage of correct IgM results was 62% [2]. The proportion of cross-reactive and inconclusive results was higher among the IgM assays, and this, we assume, occurred mainly due to weak or at-the-threshold signal in pooled commercial assays. Fortunately, most of the participating laboratories that performed these pooled assays, also used confirmatory assays, such as IB or IFA, and this finally led to accurate result of panel samples (see Diagnostic Conclusions). In summary, compared with the previous EQAs, there has been considerable improvement in the diagnosis of acute orthohantavirus infections via serological assays. However, due to relatively low numbers of participating laboratories, the findings cannot be extrapolated directly to the larger diagnostic community.

A high proportion of the assays used for orthohantavirus IgG detection were commercial assays, not designed to differentiate between the different orthohantavirus species. However, in-house assays were used more often for IgG detection than for IgM detection. Although percentages of false-negative results and cross-reactive results were lower in IgG assays than IgM assays, the number of total correct positive results was also lower than for IgM assays. The main reason for this is that IgM negative and IgG positive samples were slightly reactive in IgM assays, which led some laboratories to report their final conclusions incorrectly for past infection samples as "inconclusive”, thus decreasing the rate of accuracy of past infection serology in general. Furthermore, while the percentage of correct results for IgG in this EQA is 89%, it was 88% in the previous EQA in 2010–2011 [2]. Consequently, there has been no apparent change in the rate of IgG detection of orthohantaviruses in European expert laboratories over the past decade. Furthermore, the participating laboratories, performing orthohantavirus and LCMV diagnostics, were few. Moreover, not all participants opted to receive both molecular and serology panels, outlining differences in the array of diagnostics methods available across Europe.

## Conclusion

External quality assessments are useful for assessment diagnostic capability and accuracy, and it is important to perform EQAs periodically. Here we targeted methods to detect and diagnose rodent-borne virus infections. This EQA demonstrated that serological diagnosis, which comprises almost all diagnostics used for acute human orthohantavirus infections occurring in Europe, is at an acceptable level with 95% accuracy. However, the performance of molecular detection methods for use in clinical diagnostics of orthohantaviruses could be improved, for example through introduction and use of S-segment targeted or orthohantavirus species specific assays. While molecular methods to detect LCMV were performing well, the number of participating laboratories with this capacity was limited.

## Supplementary Data

241000 Supplementary Material
