# Supplementary material for: External quality assessment of orthohantavirus and lymphocytic choriomeningitis virus molecular detection and serology in Europe, 2021
Source: Euro Surveill. 2023 Oct 5;28(40):2300054. doi: 10.2807/1560-7917.ES.2023.28.40.2300054 (PMC10557384; doi:10.2807/1560-7917.ES.2023.28.40.2300054)
Supplement: Supplementary Material [file 23-00054_VAPALAHTI_Supplement.pdf]

This supplementary material is hosted by *Eurosurveillance* as supporting information alongside the article "External quality assessment of orthohantavirus and lymphocytic choriomeningitis virus molecular detection and serology in Europe, 2021", on behalf of the authors, who remain responsible for the accuracy and appropriateness of the content. The same standards for ethics, copyright, attributions and permissions as for the article apply. Supplements are not edited by *Eurosurveillance* and the journal is not responsible for the maintenance of any links or email addresses provided therein.

Supplementary Table S1. Results and detailed methods used for detection of orthohantaviruses and lymphocytic choriomeningitis virus in the molecular panel of the external quality assessment, Europe, 2021

Method	Number of laboratories	Target	Correct positive results[a]	False negative[a]	False positive[a]	Inconclusive[a]
Orthohantaviruses						
Klempa et al., 2006 [15]	5	Pan-Hanta L-Segment	9/35	26/35	0/60	1/60
Aitichou et al., 2005 [12]	1	Hantavirus S-Segment	5/7	2/7	0/12	0/12
Weidmann et al., 2005 [16]	1	DOBV S-Segment	1/2	0/2	0/12	1/12
Kramski et al., 2007 (Real-Time RT-PCR) [13]	4	Hantaviruses (Separate PCR sets) S-Segment	13/13	0/13	0/48	1/48
Kramski et al., 2007 (Modified conventional RT-PCR) [13][b]	1	Hantaviruses	7/7	0/7	0/12	2/12
Bowen et al., 1997 [17]	1	PUUV/TULV S-Segment	0/2	2/2	0/12	1/12
Pang et al., 2014 [18]	1	Hantavirus S segment	6/7	1/7	1/12	0/12
Illumina NextSeq Kit	1	Unbiased HTS approach	3/7	4/7	0/12	0/12
FastTrack Diagnostics	1	HTNV	0/1	1/1	2/12	0/12
Papa et al., 1998 [19]	1	DOBV/SEOV S segment	4/4	0/4	0/12	0/12
Niskanen et al., 2019 [20]	1	PUUV S segment	2/2	0/2	0/12	0/12
Altona	1	Hantavirus (Unknown)	5/7	2/7	0/12	0/12
Unknown[c]	1	HTNV[d]	1/1	0/1	0/12	0/12
	1	PUUV[d]	1/2	0/2	0/12	1/12

	1	HTNV/SNV S Segment	1/1	0/1	0/12	0/12
	1	DOBV/HTNV/SEOV/PUUV	7/7	0/7	0/12	0/12
	1	Hantavirus S segment	2/7	5/7	0/12	0/12
	1	DOBV S segment	0/2	2/2	2/12	0/12
	1	PUUV S segment	1/2	½	1/12	0/12
**Total (%):**			**58.62**	**39.66**	**1.92**	**2.24**
Lymphocytic choriomeningitis virus						
Bowen et al., 1997 [21]	1	LCMV S Segment	2/2	0/2	0/12	0/12
Cordey et al., 2011 [22]	1	LCMV S segment	2/2	0/2	0/12	0/12
Vieth et al., 2007 [23]	1	Pan-Arena L segment	2/2	0/2	0/12	0/12
Fornuskova et al., 2021 [24]	1	LCMV L segment	2/2	0/2	0/12	0/12
Coulibaly-N'Golo et al., 2011 [25]	1	LCMV S segment	2/2	0/2	0/12	0/12
Sequencing	1	LCMV	2/2	0/2	0/12	0/12
Own Design (Unpublished)	1	LCMV S segment	2/2	0/2	0/12	0/12
Unknown[c]	1	LCMV[d]	2/2	0/2	0/12	0/12
	1	LCMV L segment	½	½	2/12	0/12
	2	LCMV S segment	4/4	0/4	0/24	0/24
**Total (%)**			**95.45**	**4.55**	**1.52**	**0**

DOBV: Dobrava-Belgrade orthohantavirus; HTNV: Hantaan orthohantavirus; HTS: High-throughput sequencing; LCMV: lymphocytic choriomeningitis virus; PUUV: Puumala orthohantavirus; SEOV: Seoul orthohantavirus; TULV: Tula orthohantavirus.

[a]'Correct Positive Results' and 'False Negative' were calculated by taking into account the number of panel samples containing the target virus. In contrast, 'Inconclusive Results' and 'False Positive' were calculated using the total number of samples (12) that the molecular EQA panel contained.

[b]An inconclusive result with this method was for one of the negative samples.

[c]Laboratories that did not provide any protocol for molecular detection were classified under 'Unknown'

[d]Known target virus, but unknown target segment/ORF/gen

Supplementary Table S2. Results and detailed methods for determination of acute and past infection of orthohantaviruses in the serology panel of the external quality assurance, Europe, 2021

Method	Reference	Target	Number of laboratories	Target result	Correct results[a]	False negative Result[a]	Cross-Reactive Results[a,b]	Cross-Reactive Virus[a]	Inconclusive Results[a]
Acute infection (IgM)									
EIA	Reagena	PUUV	1	1	1	0	0/4	None	0/4
	Lee et al., 1998 [26]	PUUV-SNV-THAIV	1	2	2	0	1/4	DOBV(IgG) +	0/4
	Euroimmun	Anti-Hantavirus Pool	8	16	16	0	5/32	DOBV(IgG) +	8/32
	FOCUS Diagnostics	Hantavirus	2	4	3	1	3/8	DOBV(IgG) + ; PUUV (IgG) +	0/8
	Abcam	Hantavirus Generic	1	2	2	0	0/4	None	1/4
ICA	Reagena	DOBV-HTNV	2	2	2	0	1/8	PUUV(IgM) +	1/8
		PUUV	2	2	2	0	2/8	DOBV(IgM) +	1/8
IB	Mikrogen	Hantavirus IgM	3	6	6	0	0/12	None	1/12
	Euroimmun	Anti-hanta Profile 1	3	6	6	0	1/12	PUUV(IgG) +	3/12
IFA	in-house	DOBV-HTNV-PUUV-SEOV	1	2	2	0	0/4	None	0/4
	Euroimmun	Hantavirus mosaic	3	6	4	0	3/12	DOBV(IgG) + ; PUUV(IgG) +	2/12
**Total (%):**					**93.87**	**2.04**	**14,81**		**15.74**
Past infection (IgG)									
EIA	Reagena	PUUV	1	2	2	0	0/4	None	0/4
	Rossi et al., 1998	PUUV-SNV-	1	2	2	0	2/4	DOBV (IgM,	0/4

	[25]	THAIV						IgG) +	
	Euroimmun	Anti-Hantavirus Pool	9	36	33	1	0/36	None	2/36
	FOCUS Diagnostics	Hantavirus	1	4	4	0	0/4	None	0/4
	Abcam	Hantavirus Generic	1	4	4	0	0/4	None	0/4
IFA	in-house	KHFV	1	2	2	0	0/4	None	1/4
		SEOV	1	0	0	0	3/4	DOBV (IgM, IgG) + ; PUUV (IgG) +	0
		PUUV	1	2	2	0	0/4	None	0/4
		DOBV-HTNV-PUUV-SEOV	1	4	3	0	1/4	PUUV(IgG) + ; DOBV (IgG)-	0/4
	Rossi et al., 1998 (Modified) [25]	PUUV-THAIV	1	2	2	0	2/4	DOBV (IgM, IgG) +	0/4
	Euroimmun	Hantavirus mosaic	3	12	8	0	2/12	HTNV(IgG) +	2/12
IB	Euroimmun	Anti-hanta Profile 1 / Profile Global	3	12	10	0	2/12	HTNV(IgG) + ; SNV(IgG) +	0/12
	Mikrogen	Hantavirus IgG	3	12	12	0	0/12	None	0/12
**Total (%):**					**89.36**	**1.06**	**11.11**		**4.63**

DOBV: Dobrava-Belgrade orthohantavirus; EIA: Enzyme Immunoassay; HTNV: Hantaan orthohantavirus; IB: Immunoblot assay; ICA: Immunochromatography assay; IFA: Immunofluorescence assay; KHFV: Korean haemorrhagic fever virus; LCMV: lymphocytic choriomeningitis virus; PUUV: Puumala orthohantavirus; SEOV: Seoul orthohantavirus; SNV: Sin Nombre virus; THAIV: Thailand virus.

[a]The 'Number of Correct Results', 'Inconclusive Results' and 'False Negative Results' were calculated using only the total number of target samples (n = 91). In contrast, 'Cross-Reactive Results' was calculated by including all samples that might potentially give a positive result (n = 108; Number of submissions x Number of potentially reactive samples for one submission = Total number of potentially reactive samples).

[b]These cross-reactive results were either heterologous detections of different sero-group orthohantaviruses or between acute infection (IgM + /IgG +) and past infection (IgM-/IgG +) samples

Supplementary Table S3. Nucleic acid extraction methods (n=16) used in this EQA for molecular detection of orthohantaviruses and LCMV, Europe, 2021.

Extraction Type	Extraction Method	Number of Laboratories
Automated	MagMAX Viral/Pathogen NA isolation kit (Thermo Fisher, Waltham)	1
	Elitech InGenius (ELITech Group, Puteaux)	1
	MagLEAD 6gC/12gC (PSS, Tokyo)	1
	Maxwell RSC 48 Viral Total NA kit (Promega, Charbonnières-les-Bains)	1
	QIAamp Viral RNA Mini QIAcube kit (Qiagen, Hilden)	2
	MagNA Pure 96 DNA and Viral NA kit (Roche, Meylan)	2
	MagNA Pure LC Total NA kit (Roche, Meylan)	1
	MagPurix Viral/Pathogen NA Extraction kit A (Zinexts Life Science, New Taipei City)	1
Manual	Nucleospin Dx Virus kit (Macherey-Nagel, Dueren)	1
	QIAamp viral RNA mini kit (Qiagen, Hilden)	4
	GeneJet Viral RNA purification kit (Thermo Fisher, Waltham)	1
